# Prolonged Therapy with Imatinib Mesylate before Surgery for Advanced Gastrointestinal Stromal Tumor Results of a Phase II Trial

**DOI:** 10.1155/2012/761576

**Published:** 2012-12-17

**Authors:** C. Doyon, L. Sidéris, G. Leblanc, Y. E. Leclerc, D. Boudreau, P. Dubé

**Affiliations:** Department of Surgery, Maisonneuve-Rosemont Hospital, University of Montreal, Montreal, QC, Canada H1T 2M4

## Abstract

*Purpose*. Proven efficacy of imatinib mesylate in gastrointestinal stromal tumour (GIST) has led to its use in advanced disease and, more recently, in adjuvant and neoadjuvant settings. The purpose of this study was to evaluate the optimal neoadjuvant imatinib duration to reduce the morbidity of surgery and increase the possibility of resection completeness in advanced tumours. *Patients and Method*. Patients with advanced GIST were enrolled into a registered open-label multicenter trial and received imatinib daily for a maximum of 12 months, followed by en bloc resection. Data were prospectively collected regarding tumour assessment, response rate, surgical characteristics, recurrence, and survival. *Results*. Fourteen patients with advanced GIST were enrolled. According to RECIST criteria, 6 patients had partial response and 8 had stable disease. The overall tumour size reduction was 25% (0–62.5%), and there was no tumour progression. Eleven patients underwent tumour resection, and all had R0 resection. After a median followup of 48 months, 4-year OS and DFS were 100% and 64%, respectively. *Conclusion*. This prospective trial showed that one year of neoadjuvant imatinib in advanced GIST is safe and associated with high rate of complete microscopic resection. It is not associated with increased resistance, progression, or complication rates.

## 1. Introduction

Gastrointestinal stromal tumour (GIST) is the most common mesenchymal malignancy of the gastrointestinal tract [1]. After providing evidence that GIST has a distinct identity by sharing features with the interstitial cells of Cajal [2], encoding gain-of-function mutations were found on the c-kit and platelet-derived growth factor-alpha (PDGFR-*α*) genes [3, 4], now known as hallmarks in tumorigenesis of GIST [5–7].

Surgery has historically been the only curative option for GIST, but long-term survival rates were low, with about 20% of patients alive at 10 years [8]. In advanced GIST, best supportive care was the only option in most cases due to general resistance to conventional chemotherapy [9].

Imatinib mesylate (Gleevec; Novartis, Basel, Switzerland) [10] (IM), a tyrosine kinase inhibitor of the constitutional activity of c-kit and PDGFR-*α* [11], has revolutionized the management of unresectable, recurrent, and metastatic GIST, with significant improvement on progression-free survival (PFS) and overall survival (OS) [12–16]. In the last five years, there has been a growing interest about the potential advantages of IM in high-risk and potentially curable diseases. Recently, treatment recommendations from the European Society for Medical Oncology (ESMO) and the National Comprehensive Cancer Network (NCCN) added the option of adjuvant IM for patients at high risk of relapse [17, 18], based primarily on recurrence-free survival (RFS) gains obtained in the ACOSOG Z9001 trial [19]. Moreover, based on preliminary results of the Scandinavian 3-year versus one-year adjuvant study presented at the american society of clinical oncology (ASCO) 2011 [20], the final ASCO recommendations mention that the standard duration of adjuvant treatment for high-risk local GIST should be 3 years. 

On the other hand, many questions regarding the use of IM in advanced GIST remain unanswered. Numerous retrospective studies have supported the use of preoperative IM to downsize large tumours in order to decrease the risk of perioperative complications and increase the feasibility of surgery [21–29]. Furthermore, two recent prospective phase II studies using IM as neoadjuvant and adjuvant treatment in potentially resectable GIST reported promising results regarding safety, tolerability, and tumour response [30, 31]. In both of these studies, preoperative IM was used for a maximum of 12 weeks, and one may question whether maximal tumour response had been achieved prior to surgery, since there is evidence in the literature that tumour response may take several months to occur in advanced disease [32].

This prospective phase II trial was designed to evaluate the efficacy of prolonged preoperative IM on the microscopically complete resection rate (R0) of potentially resectable advanced GIST and to observe whether its prolonged use achieved better maximal tumour response. 

## 2. Methods

GAP study was a phase II registered (NCT00290485) prospective, one-arm multicenter trial. Two affiliated centers were involved in the study. In addition to usual eligibility criteria, patients must have had positive kit receptor marker and locally advanced or metastatic, but potentially resectable, GIST. Eastern Cooperative Oncology Group performance score was recorded for each patient. Advanced GIST was defined as a tumour with a significant risk of non-R0 resection, as judged on the imaging. These tumours could have been adherent to significant intra-abdominal organs (e.g., liver, kidney, oesophagus, rectum, etc.) or display a single resectable metastatic site. Exclusion criteria were prior systemic chemotherapy, radiotherapy, and/or IM treatment.

After signing informed consent, patients started IM at a dose of 400 mg daily. In the case of no radiological response at week 7, IM was escalated to a daily dose of 600 mg in order to achieve a better response without added toxicity based on the experience of an open-label, multinational study that was conducted in patients with unresectable or metastatic malignant GIST [13, 33, 34]. Furthermore, we did not use the dose of 800 mg per day for progressing tumor and for exon 9 mutation tumor. In fact, by the time the study was designed and achieved, SWOG [33] and EORTC [14] studies were not published yet. Also, neither of those two studies succeeded to demonstrate an advantage on overall survival in further meta-analysis [35, 36]. After week 9, if progressive disease was seen on radiological examination, laparotomy was performed when feasible. Initially, the preoperative treatment duration was planned for 6 months. However, based on the results published by Haller et al. regarding maximal tumour response and IM treatment duration [32], an amendment was brought to the study after the first 3 patients, and the duration of neoadjuvant IM was increased to 12 months, except in cases of disease progression or complications during treatment. Clinical assessment and complete blood count were performed every 4 weeks until surgery. Adverse event severity was graded according to the National Cancer Institute Common Toxicity Criteria, version 2.0 [37]. Radiologic (CT scan or MRI) response was evaluated according to RECIST criteria [38] at weeks 7, 15, 23, 31, 39, and 47. The same imaging modality was used once started with a patient. IM was stopped one day prior to surgery. RECIST criteria were chosen as the more relevant criteria to be used for tumor assessment of GIST as shown in validations studies [39]. Also, by the time of the study design, the publication of Choi and his colleagues was not published yet [40].

Surgical specimens were assessed for c-kit and PDGFR-*α* mutation, size, margins, necrosis, and rate of mitosis/50 high power field (HPF) after rapid fixation with formalin.

Surgical complications were graded according to Dindo's classification [41]. The first followup visit occurred one month after discharge and every 3 months thereafter with clinical and radiological assessment. Postoperative IM treatment was left at the surgeon's discretion. In fact, the first patients included in the study were treated before the publication of ACOSOC Z9000 that proved the efficacy of IM for one year after surgery in local resectable GIST [42].

Time to recurrence and RFS were both measured from date of surgery. Overall survival (OS) was measured from the beginning of IM treatment. Kaplan-Meier's survival curves was established for RFS probabilities [43]. Data were analyzed using SPSS version 11.0 for Mac OS X. 

## 3. Results

A total of fifteen patients were accrued in the study between July 2004 and April 2007. Our goal to include 50 patients in the study was not achieved due to the rarity of this tumor and the specificity of the population studied. One patient had to be excluded from the study due to a misdiagnosis. A total of 14 consecutive patients were included in the study. Baseline patient characteristics are provided in Table 1. The majority of patients' tumours were primary advanced GIST (*n* = 12), but 2 patients had recurrent GIST: one recurrence was local (stomach) and the other confined to the liver. Median tumour diameter at diagnosis was 9.4 cm (1.7–17.3). 

IM was started at 400 mg daily and had to be upgraded from 400 mg to 600 mg daily at week 9 in 7 patients because of nonresponse or stability according to RECIST criteria. The dosage was upgraded in stable disease in order to achieve a partial response to downsize the tumor enough to permit the surgeon to resect entirely the locally advanced GIST with negative margins. Median duration of preoperative treatment was 9 months. Nine patients (64%) completed 12 months of IM treatment. Two patients (14%) underwent surgery after 6 months of treatment (before the amendment) and 3 patients after 4, 2.4, and 2 months, respectively. Two of these patients underwent surgery before 12 months because of having a stable disease according to RECIST but progressing according to the clinician, and the other patient had to discontinue IM after 2 months because of upper gastrointestinal bleeding. 

IM was generally well tolerated with minor toxicity. One patient (7%) reported grade 3 nausea. There were 3 adverse events related to tumour bleeding and one required a semiurgent surgical resection, but these events are probably due to tumour-response instead of drug-related events [37].

**Figure 1:**
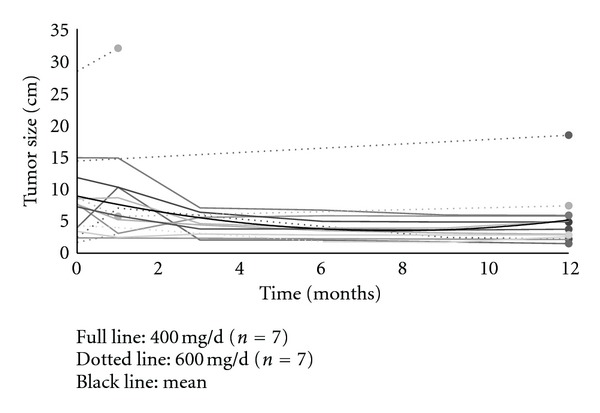
Tumour size evolution for each patient compared to mean tumour size (gray line) (*n* = 14).

**Figure 2:**
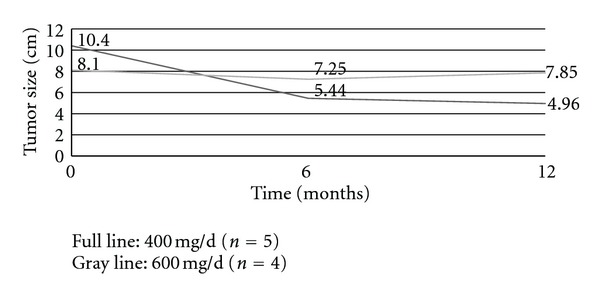
Mean tumour size in time in patients responding to neoadjuvant imatinib treated for 12 months (*n* = 9).

The evolution of clinical response to preoperative IM for each patient is reported in Figure 1, and the total response rates to preoperative IM treatment are reported in Table 2.  Figure 2 compares the evolution in time of mean tumour size for patients responding to 400 mg versus 600 mg of neoadjuvant IM treated for 12 months. According to RECIST criteria, neither had progressive disease nor complete response. Eight patients presented a stable disease, and 6 had a partial response. Three patients never had any reduction in tumour size despite that all were switched to 600 mg/d IM dosage. 

Ten of 14 patients (71%) underwent elective surgery after their preoperative treatment. One had semi-urgent surgery after 8 weeks of treatment because of gastric bleeding. The patient that presented initially in the study with a local recurrence to the stomach was found to have a nonresectable tumour intraoperatively. The patient with initial metastatic disease confined to the liver was found to be resectable after 6 months of neoadjuvant. Two patients refused surgery. Surgical strategy before and after preoperative IM treatment is detailed in Table 3.

There were no perioperative deaths, but six patients had postoperative complications. There was one grade I delirium, 3 grade II cardiac complications consisting of non-Q wave myocardial ischemia, and one pulmonary oedema, all treated with drugs only. There were 2 grade III anastomotic leaks: one after oesophagectomy that was drained in the operating room and one after total gastrectomy that was treated conservatively. 

Specimen pathological analysis revealed that all patients who underwent tumour resection (*n* = 11) had an R0 resection (negative microscopic margins). Varying amount of necrosis was present in all surgical specimens, and 3 of them (27%) showed complete tumour necrosis.

Median follow-up time following IM initiation was 50.8 months (*n* = 14), and median follow-up time following surgery was 48 months (*n* = 11). At the time of data analysis, 7 patients did not have any evidence of recurrence, 4 patients were alive with recurrence (one with ocular and pulmonary metastasis and the 3 others with stable metastatic recurrence in the liver), 1 patient was dead, and one was lost to follow-up. Median time to recurrence was 20.1 months. All patients with recurrence were still under tyrosine kinase inhibitor treatment (IM, Sunitinib, or Nilotinib). Among the 3 patients who did not undergo surgery (2 refusal and 1 unresectable), one died from a non-GIST-related cause, the one with unresectable recurrence to the stomach was lost to follow-up with progressive disease, and one presented a stable disease on IM with a median follow-up of 41 months. 

**Figure 3:**
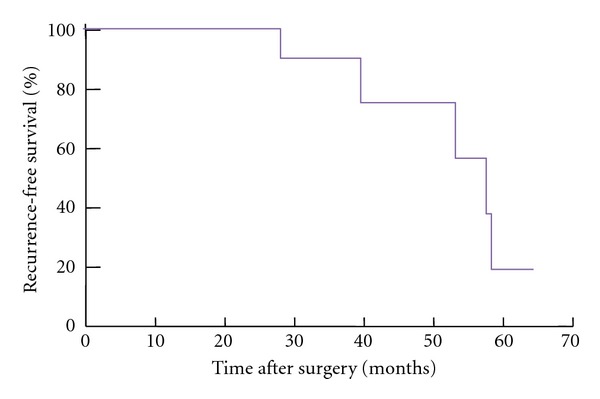
Recurrence-free survival after surgery with median F/U of 48 months (*n* = 11).

Median OS and RFS for all patients (*n* = 14) were 51 months and 44 months, respectively. Four-year OS and RFS rates for patients who underwent surgery were 100% and 64%, respectively. The estimated 5-year RFS is reported in Figure 3. 

When looking at the 7 recurrence-free patients, 4 had a partial response to preoperative IM and 3 had a stable disease. Among the 4 patients who had a recurrence, one initially presented a partial response and 3 a stable disease. The patient with the initial metastatic disease confined to the liver is one of the recurrence-free patients.

## 4. Discussion

Therapy with preoperative IM in marginally operable GIST is now recommended for consideration in both ESMO and NCCN guidelines [17, 18]. The major interest with preoperative IM in advanced GIST is its potential value as a method for tumour downsizing, where the goal is a less extensive surgery with less complications and a more complete resection [44]. Despite two recently published prospective trials using preoperative IM in advanced resectable GIST [30, 31], to our knowledge, there have been no prospective reports using preoperative IM until maximal tumour response had been reached (between 6 and 12 months) [32]. The use of preoperative IM for twelve weeks or less is now proven to be safe and feasible with few adverse effects. It is also associated with tumour downsizing, necrosis, and a decrease in glucose uptake [30, 31]. However, studies in the metastatic setting have shown that some tumours may respond after more than 12 months of IM therapy [13, 15], and this was the main rationale justifying prolonged preoperative treatment with IM in our study. 

The rationale to administer prolonged preoperative IM is even more justified today since the presentation of the results of both the ACOSOG [19] and Scandinavian [20] studies. In our opinion, it is indicated to give preoperative IM for up to 1 year for large, advanced tumours because of the favourable toxicity profile of the drug and because patients with tumours as small as 3 cm benefited from one year of adjuvant IM in the ACOSOG study [19]. Furthermore, the preliminary results of the Scandinavian study (1 versus 3 years of adjuvant IM), presented at the American society of clinical oncology (ASCO) 2011 [20], showed that all subgroups benefit from the prolonged IM treatment. The final ASCO recommendations mention that the standard duration of adjuvant treatment for high-risk local GIST should be 3 years. 

Despite the small number of patients, this study showed that the administration of preoperative IM for at least 6 months in marginally operable GIST patients should be considered. It is safe and effective, and it decreases the extent of surgery and possibly surgical morbidity. It has also resulted in a high rate of complete microscopic resection (R0) in marginally resectable local or metastatic GIST. This could be explained by the fact that prolonged duration of preoperative IM administration (2.1 months versus 6 to 12 months in our study) induced a greater decrease in tumour size preoperatively and allowed for less morbid surgery (as shown by Tables 2 and 3), with better chances to obtain negative margins. 

It is noteworthy that in our study, patients who did not initially respond to IM 400 mg daily did not seem to benefit more from increasing the dose to 600 mg. In responders, the best maximal response was obtained at 6 months. There was no deleterious effect either on toxicity or resistance to IM to prolong preoperative IM for 12 months before undergoing surgery, as also shown in the ACOSOG [19] and Scandinavian adjuvant studies [20]. The beneficial effect of the molecule on patient survival and resection rates is probably worth the cost of the mild toxicities induced by prolonged IM therapy. 

As already discussed by Gold and DeMatteo [45], it is very difficult to prove that preoperative treatment improves resectability. This was the case in our study, since some patients still had to undergo high-risk surgery even with a good preoperative response. Nonetheless, our study showed that only 36% of patients had multiple organ resections. There were no perioperative deaths, and the 18% rate of postoperative surgical complications is acceptable considering the extent of surgery.

The timing of surgery for patients being treated with preoperative treatment is still debated by many authors and remains unclear. Some advocate the fact that giving IM for a longer period could predispose to secondary c-kit mutations, allowing for disease progression due to molecular evolution of resistant clones, thus decreasing the patient's chance to be amenable for surgery and prognosis [29]. Others give around 6 months of treatment before surgery [46, 47], based on cohort studies that have shown that maximum tumour response to IM occurs between 2.7 and 9 months [14, 15]. Haller and colleagues [32] have suggested operating as short as curative tumour resection without morbid surgery or function-sparing surgery can be carried out, and we agree with this. Our results have demonstrated that, like Gronchi and colleagues proposed [21], 6 to 12 months (average 9 months) of preoperative treatment achieved the best tumour response and did not seem to induce secondary resistance/progression in patients during the course of treatment. 

 When looking at the 4 patients who presented a recurrence, all initially had an R0 resection of their tumour. It supports the fact that other factors are to be considered to predict patterns of recurrence. Tumour mitotic rate, size and location of the tumour, c-kit codons 557 to 559 exon 11 deletion, exon 13 and 17 mutations, and intraoperative tumour capsule rupture have all recently been proven to be independent predictors of recurrence [48–51]. Future adjuvant and neoadjuvant trials should take these factors into consideration. 

As stated in the recent literature [52–55], we also think that RECIST criteria are probably not the best to evaluate GIST response to IM. Two patients who presented a stable and partial disease, respectively, with RECIST criteria revealed to have a complete pathologic response with no viable tumour measurable on surgical specimens at final analysis. Another patient, who had a stable tumour according to RECIST with a slight increase in tumour volume, showed partial pathological response with over 30% of necrosis. These results support the idea of adding new criteria like tumour density in the evaluation of IM response as proposed by Choi et al. [52].

This prospective study had a relatively small group of patients, which is not unique to our study with such a rare disease. However, it is the only multi-institutional trial that has addressed the question of preoperative IM for a prolonged period until maximal tumour response has been reached. With a median postoperative follow-up of 48 months, survival results are mature. In marginally resectable GIST, we recommend at least 6 months of preoperative IM to all patients. If the patient does not respond to IM or if bleeding occurs, laparotomy should be the best therapeutic option at any time. This approach is feasible and safe, and it is not associated with increased resistance, progression, or complication rates. A phase III study with larger patient population would be necessary to confirm our primary results. APOLLON, a prospective study determining the best duration and efficacy of neoadjuvant treatment, is now close, and preliminary results presented at ASCO 2012 are promising concerning the safety of 6 months of preoperative IM on reducing the extent of the operation and on disease-free survival [56].

## Figures and Tables

**Table 1:** Patients and tumour characteristics (*n* = 14).

Characteristics	Number (range)
Mean age (years)	64 (39 to 84)
Gender (male/female)	
Male	12
Female	2
Tumour diameter (cm)	9.4 (1.7 to 29)
Disease status at diagnosis	—
Locally advanced	12
Local recurrence	1
Metastatic	1
Performance status at diagnosis (ECOG)	—
0	10
1	2
2	2
Disease location	—
Stomach	9
Duodenum	2
Liver	1
Oesophagus	1
Rectum	1

**Table 2:** Response to neoadjuvant imatinib (NAI).

Patient	Tumour site	Largest tumour diameter Before NAI (cm)	Largest tumour diameter After NAI (cm)	Change in tumour size (%)	Duration of NAI (months) and final dosage of imatinib (mg)	RECIST response
1	Rectum	2.5	2.2	**−12.0**	12 (600)	SD^i^
2	Stomach	8.3	5.9	**−28.9**	12 (600)	PR^ii^
3	Stomach	4	1.5	**−62.5**	6 (400)	PR
4	Duodenum	13	6.4	**−50.8**	12 (400)	PR
5	Stomach	16.5	7	**−57.6**	12 (400)	PR
6	Stomach	8.7	3.5	**−59.8**	12 (400)	PR
7	Stomach	17.3	20.2	**16.8**	12 (600)	SD
8	Stomach	9.1	10	**9.9**	2 (600)	SD
9	Liver	1.7	1.3	**−23.5**	4 (600)	SD
10	Oesophagus	9.2	5.6	**−39.1**	12 (400)	PR
11	Duodenum	2.8	2.3	**−17.9**	12 (400)	SD
12	Stomach	4.3	3.2	**−25.6**	12 (600)	SD
13	Stomach	29	34	**17.2**	2.4 (600)	SD
14	Stomach	5.6	4.5	**−19.6**	6 (400)	SD

M^iii^	—	9.4	7.7	**−25.2**	9	SD

^i^SD: stable disease (less than 30% reduction or 20% increase in largest diameter).

^ii^PR: partial response (30% reduction or more in largest diameter).

^iii^M: mean.

**Table 3:** Surgical strategy before and after neoadjuvant imatinib, resection performed, and resection margins.

Patient	Number of organs planned to be resected before NAI^i^ (details)	Number of organs planned to be resected after NAI (details)	Changes in resection due to NAI	Number of organs resected	Resection margins
1	1^ii^ (with colostomy)	1^iii^ (no colostomy)	↓	1	R0
2	1 (STG^iv^)	1 (WG^v^)	↓	1	R0
3	1 (STG)	1 (WG)	↓	1	R0
4	4 (Whipple, RC^vi^, kidney)	3 (Whipple, RC)	↓	3	R0
5	3 (STG, SP^vii^, and PA^viii^)	3 (WG, SP, and PA)	↓	3	R0
6	1 (antrectomy)	1 (antrectomy)	=	0^ix^	NA^x^
7	2 (stomach + liver)	2 (stomach + liver)	=	0^xi^	NA
8	2 (STG + left liver)	2 (STG + left liver^xii^)	=	2	R0
9	1 (liver)	1 (liver)	=	1	R0
10	4 (oesophagectomy + trachea + pericardia + vertebral body)	1 (oesophagus)	↓	1	R0
11	2 (Whipple)	2 (Whipple)	=	0^xiii^	NA
12	2 (STG + PA)	1 (STG)	↓	1	R0
13	3 (STG + SP + PA)	3 (STG + SP + PA)	=	3	R0
14	1 (antrectomy)				

^i^NAI: neoadjuvant imatinib.

^ii^Abdominoperineal resection.

^iii^Low anterior resection with primary anastomosis.

^iv^STG: subtotal gastrectomy.

^v^WG: wegde gastrectomy.

^vi^RC: right hemicolectomy.

^vii^SP: spleen.

^viii^PA: pancreas.

^ix^Patient refused laparotomy.

^x^Not applicable.

^xi^NR: nonresectable due to multiple liver metastasis.

^xii^Semielective procedure for gastric bleeding.

^xiii^Patient refused Whipple procedure (segmental duodenal resection was not possible).
